# Clinical Characteristics in the Prediction of Posttreatment Survival of Patients with Ovarian Cancer

**DOI:** 10.1155/2022/3321014

**Published:** 2022-05-05

**Authors:** Li Lu, Shuqi Ji, Jing Jiang, Yu Yan

**Affiliations:** Department of Obstetrics and Gynecology, 2nd Affiliated Hospital of Harbin Medical University, China

## Abstract

**Objective:**

To determine the efficacy of clinical characteristics in the prediction of prognosis in patients with ovarian cancer.

**Methods:**

Clinical data were collected from 3 datasets from TCGA database, including 1680 cases of ovarian serous cystadenocarcinoma, and were analyzed. Patients with ovarian cancer admitted to our hospital in 2016 were retrieved and followed up for prognosis analysis.

**Results:**

From the datasets, for patients > 75 years old at the time of diagnosis, histologic grade and mutation count were good predictors for disease-free survival, while for patients > 50 years old at the time of diagnosis, histologic grade, race, fraction genome altered, and mutation count were good predictors for overall survival. In the patients (*n* = 38) retrieved from our hospital, the longest dimension of lesion (cm) and body weight at admission were good predictors for overall survival.

**Conclusions:**

Those clinical factors, together with the two predictive equations, could be used to comprehensively predict the long-term prognosis of patients with ovarian cancer.

## 1. Introduction

Ovarian cancer is the third most common as well as the fifth cause of deaths of gynecologic cancers. The American Cancer Society estimates that in 2022, about 19,880 women will be newly diagnosed of ovarian cancer and about 12,810 women will die from it [1].

Patients with ovarian cancer have different clinical characteristics and prognosis. Based on data collected between 2010 and 2016, only about 20% of ovarian cancers were diagnosed at an early stage, which had a 5-year survival rate of 94%. In contrast, the 5-year relative survival rate of all SEER (Surveillance, Epidemiology, and End Results) stages combined invasive epithelial ovarian cancer patients was only 48%, with localized ones being 93% and distant ones being 31% [2]. Therefore, more efforts are needed to accurately predict the prognosis in later stage ovarian cancer in order to find clues to improve the prognosis.

In this study, we investigated into the clinical characteristics that could be used to effectively predict the prognosis of patients with ovarian cancer.

## 2. Materials and Methods

### 2.1. Patient Sources

Patient clinical data were obtained from the TCGA database (https://cancergenome.nih.gov), including the Firehose Legacy dataset (*n* = 606), the Nature 2011 dataset (*n* = 489), and the PanCancer Atlas dataset (*n* = 585). All cases were included for analysis when the corresponding parameter was available. Patients diagnosed with primary ovarian cancer admitted to our hospital from January 1, 2016, to December 31, 2016, were retrieved and followed up for prognosis analysis. The retrospective portion of this study was approved by our hospital's ethical committee, and informed consents were obtained from the enrolled patients or their family member (if the patients died) during follow-up contact.

### 2.2. Data Extraction

Two authors independently extracted data and confirmed the accuracy of data. Clinical characteristics, such as age at diagnosis, disease-free survival, overall survival, clinical stages, histologic grades, race, fraction genome altered, Karnofsky performance score, longest dimension of lesion, lymphovascular invasion indicator, primary tumor site, neoplasm status, and mutation count were extracted from the above database when available. Due to the inconsistency in data collection among datasets, the exact number of patients in each parameter might be different.

### 2.3. Statistical Analyses

Statistical analyses were carried out by a third author. Measurement data were shown as mean ± standard deviation (SD). The Kaplan-Meier survival curve was used to analyze the associations between clinical characteristics and prognosis, including disease-free survival and overall survival. Receiver operating characteristic (ROC) curve was used to illustrate the predictive value of clinical characteristics on 5-year survival. Predictive equation for 5-year survival based on clinical factors was obtained using multinomial logistic regression. All statistical analyses were carried out using SPSS 24.0 (SPSS Inc., Chicago, USA). A *p* value < 0.05 (two-sided) was considered statistically significant.

## 3. Results

### 3.1. Clinical Characteristics of Enrolled Patients

There were 1692 patients retrieved from the three TCGA database (Table 1). The mean age at diagnosis was 59.6 years old. Disease-free survival and overall survival were shown using mean ± SD. Clinical stages, histologic stages, race, fraction genome altered, longest dimension of lesion, primary tumor site, and mutation count were further analyzed in corresponding subgroups. There were 38 cases with available data during our follow-up contact, with an average age of 49.8 ± 14.4 years old.

### 3.2. Value of Clinical Predictive Factors for Disease-Free Survival

**Figure 1:**
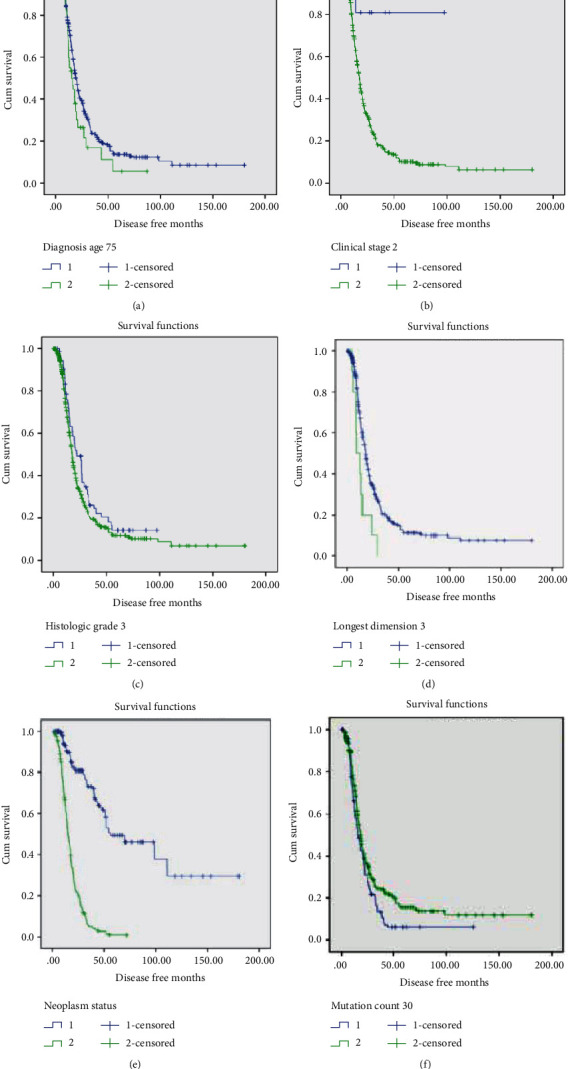
Kaplan-Meier survival curve of clinical factors for disease-free survival. (a) Age > 75 years old. (b) Clinical stage over II. (c) Histologic grade over III. (d) Longest dimension of lesion (>3 cm). (e) Neoplasm status (with tumor). (f) Mutation count (>30).

**Figure 2:**
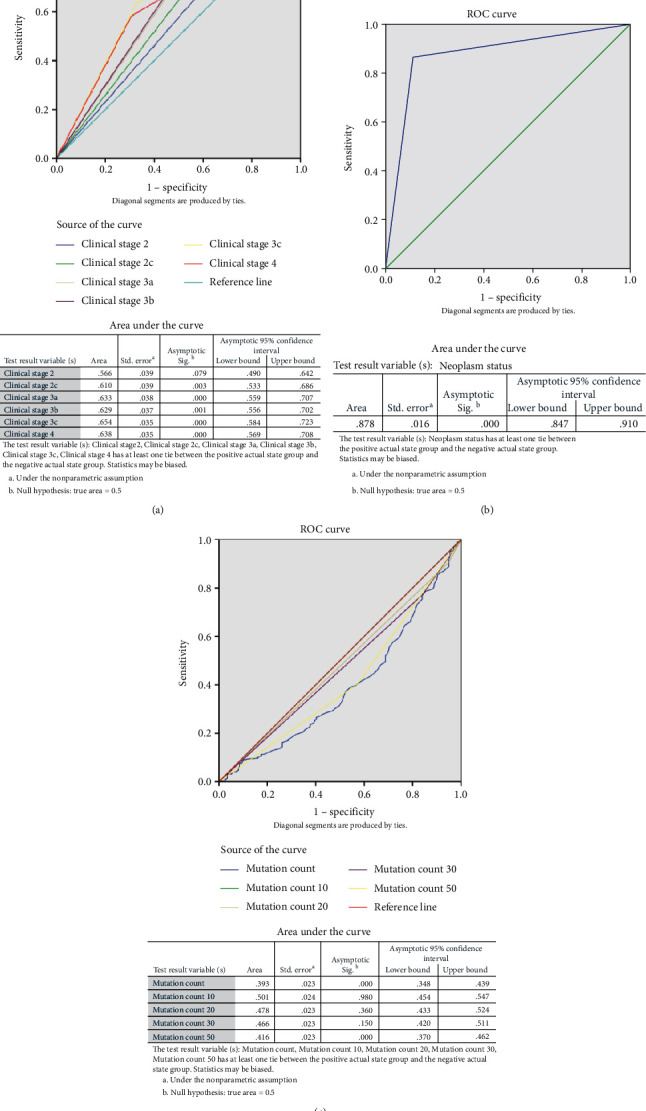
ROC curve of predictive value of clinical factors for disease-free survival. (a) Clinical stage. (b) Neoplasm status (with tumor). (c) Mutation count.

Age at diagnosis > 75 years old (*p* = 0.021), clinical stages (*p* < 0.01 for overall and subgroups), histologic stage (*p* = 0.01 for overall and and *p* = 0.014 for stage III), longest dimension of lesion >3 cm (*p* = 0.007), neoplasm status (*p* < 0.001), and mutation count (*p* = 0.004 when >30 and *p* < 0.001 when >50) were significantly associated with disease-free survival (Table 2). The Kaplan-Meier survival curves and ROC curves of corresponding factors are shown in Figures 1 and 2, respectively. According to the area under the curve, neoplasm status showed the best value (0.878) in prediction of long-term disease-free survival.

### 3.3. Value of Clinical Predictive Factors for Overall Survival

**Figure 3:**
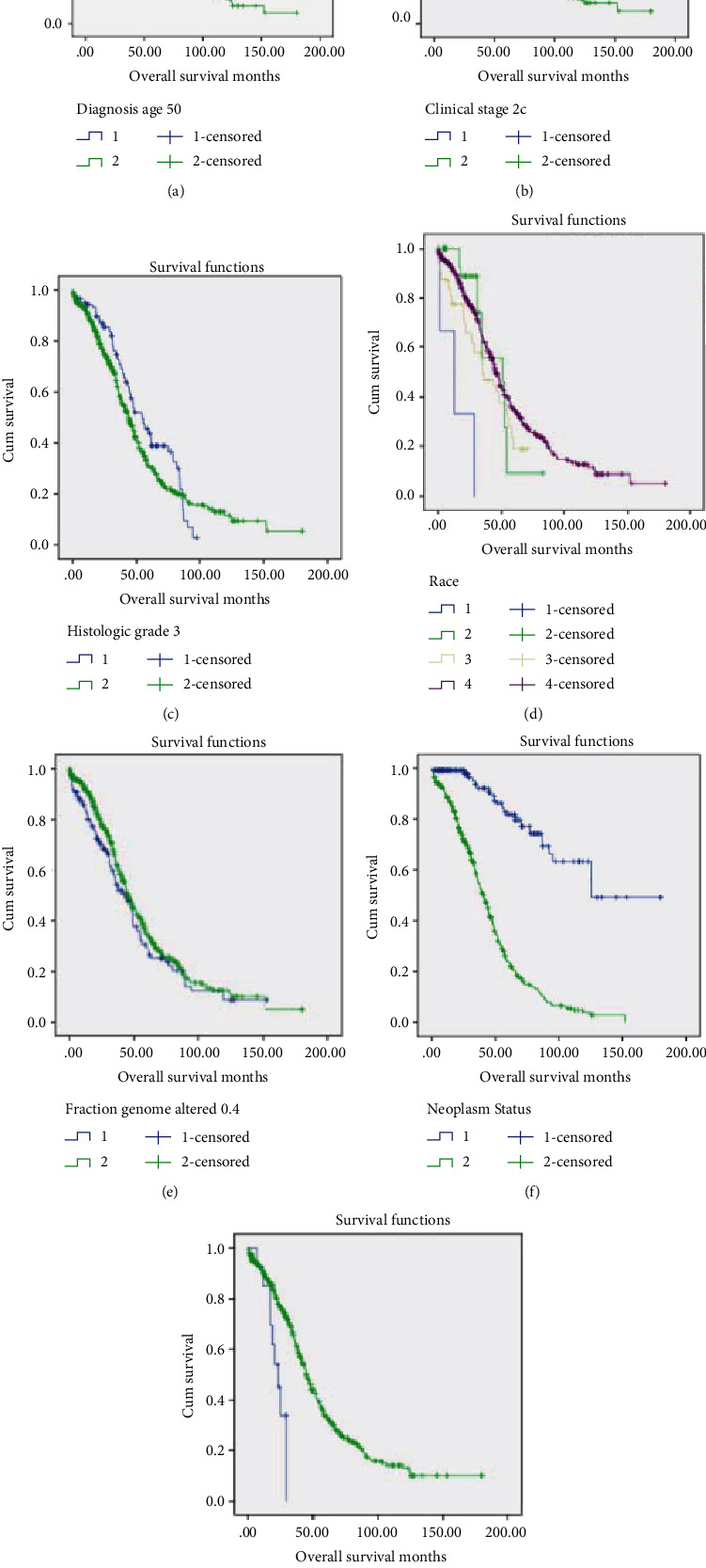
Kaplan-Meier survival curve of predictive factors for overall survival. (a) Age > 50 years old. (b) Clinical stage over IIC. (c) Histologic grade over III. (d) Race (1 = American Indian or Alaska Native, 2 = Asian, 3 = black, and 4 = white). (e) Fraction genome altered (>0.4). (f) Neoplasm status (with tumor). (g) Mutation count (>10).

**Figure 4:**
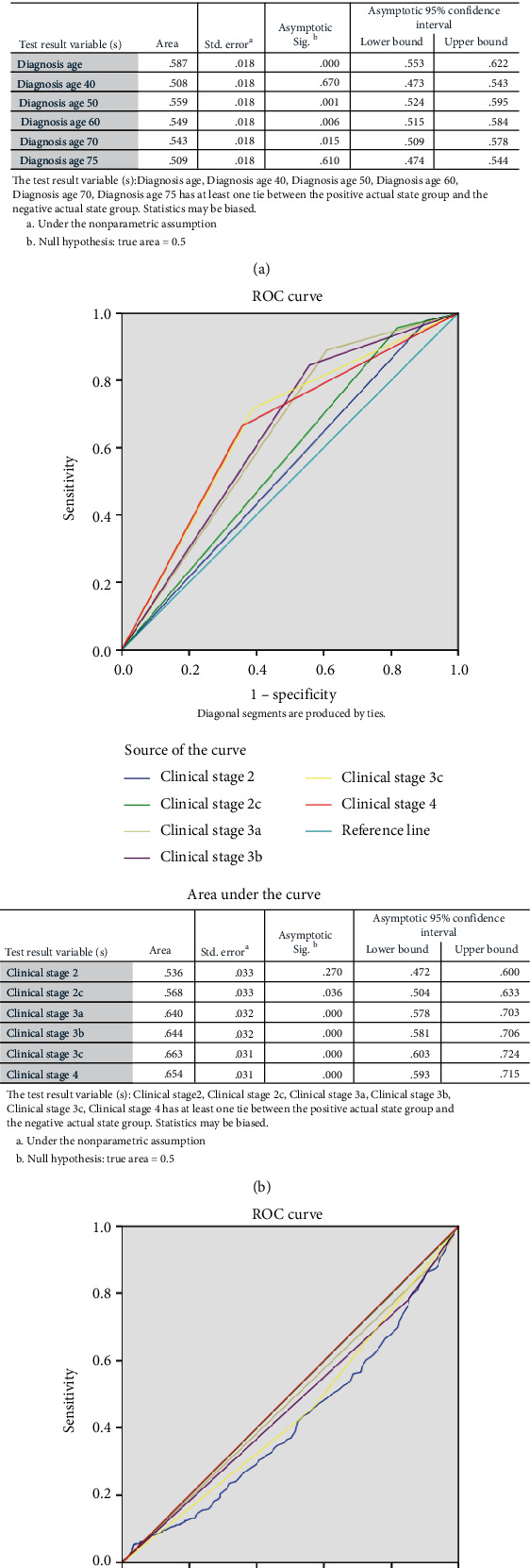
ROC curve of predictive value of clinical factors for overall survival. (a) Age. (b) Clinical stage. (c) Mutation count.

Age at diagnosis (*p* < 0.01 for overall and subgroups), clinical stages (*p* = 0.017 for IIC and *p* < 0.01 for IIIA and above), histologic grade (*p* = 0.009 for overall and *p* = 0.037 for grade III), race (*p* = 0.004), fraction genome altered (*p* = 0.032 for the 0.4 and above group, *p* = 0.03 for the 0.5 and above group, and *p* = 0.001 for the 0.6 and above group), neoplasm status (*p* < 0.001), and mutation count (*p* < 0.001 for all subgroups) were significantly associated with overall survival (Table 3). The Kaplan-Meier survival curves and ROC curves of corresponding factors are shown in Figures 3 and 4, respectively. According to the area under the curve, clinical stage above III showed the best value (>0.64) in prediction of long-term overall survival. In the patients (*n* = 38) retrieved from our hospital, the longest dimension of lesion (cm, *p* = 0.001) and body weight at admission (*p* < 0.001) were good predictors for overall survival (Table 4).

### 3.4. Predictive Equations for Disease-Free and Overall Survival

In order to obtain a more practical way to predict the prognosis and to test if all factors based on Kaplan-Meier survival curves and ROC curves are good predictors for prognosis, a predictive equation for disease-free survival based on clinical factors was obtained using multinomial logistic regression: log[p/(1 − p)] = 18.972 − 14.568 Longest Dimension of lesion–3.593 Neoplasm Status where *p* is the probability of death within 5 years, Longest Dimension of lesion = 2 if >3 cm and =1 if ≤3 cm, and Neoplasm Status = 2 if cancer lesion remained and =1 if cancer lesion was removed completely.

A predictive equation for overall survival based on clinical factors was obtained using multinomial logistic regression: log[p/(1 − p)] = −3.152 Neoplasm Status − 0.872 Diagnosis Age + 12.819 Mutation count where *p* is the probability of death within 5 years, Neoplasm Status = 1 if cancer lesion remained and =0 if cancer lesion was removed completely, Diagnosis Age = 2 if >50 years old and =1 if ≤50 years old and Mutation count = 2 if counted >10 and =1 if counted ≤10.

## 4. Discussion

In the present study, there were 17 patients diagnosed at clinical stage I, 57 patients diagnosed at clinical stage II, 841 patients diagnosed at clinical stage III, and 168 patients diagnosed at clinical stage IV. The majority of late stage cases showed the importance of identifying accurate predictive factors for prognosis and the possibility of improving the life expectancy and quality based on those important factors.

There have been reports of various biological prognostic biomarkers for ovarian cancer [3–7]. Interestingly, Yang et al. showed that some clinical variables were good predictors [8]. Their findings were based on TCGA OvCa cohort (*n* = 552), and they found that age (>60 years old), nodule of residual disease, tumor status, and clinical stage could significantly predict the prognosis. Our findings, based on 1692 cases from the updated TCGA OvCa cohort, showed that patients >75 years old had a significantly shorter disease-free survival, while patients >50 years old had a significantly shorter overall survival, which showed more challenges for the prognosis of patients diagnosed at a younger age. We also showed that histologic grade and mutation count were good predictors for disease-free survival, while histologic grade, race, fraction genome altered, and mutation count were good predictors for overall survival. The above parameters coincide with some recent reports [9].

Our study also showed that the total number of mutations, when considered as a whole, contributed positively to the long-term survival of ovarian cancer patients, which is consistent with previous studies including only BRCA1 or BRCA2 mutant cases [10]. The underlying mechanisms include different pathways of DNA repair, and more studies are needed when considering all patients not restricted to BRCA1 or BRCA2 mutant cases.

In the data extracted from patients admitted to our hospital, some parameters which were good predictors for prognosis in the published datasets seems to be invalid. This may be due to the smaller number of cases enrolled, the difference in race between the datasets and our own data, and the unavailability of certain parameters in our data.

With the development of big data techniques, data mining from available database has received more and more attention [11, 12]. Therefore, an updated analysis with more available datasets is beneficial in discovery of more valuable predictive factors. Due to the limitation in study design and retrospective manner of data retrieval, details in treatment methods [13, 14], psychological factors, and social-economical factors were missing from the available databases, such as anxiety or depression [15], income, nutrient conditions, and living habits, which could also contribute to the prognosis .

In summary, we showed from published datasets that for patients >75 years old at the time of diagnosis, histologic grade and mutation count were good predictors for disease-free survival, while for patients >50 years old at the time of diagnosis, histologic grade, race, fraction genome altered, and mutation count were good predictors for overall survival. On the other hand, the longest dimension of lesion and body weight at admission were good predictors for overall survival in our own retrieved data. Those clinical factors, together with the two predictive equations, could be used to comprehensively predict the long-term prognosis of patients with ovarian cancer.

## Figures and Tables

**Table 1:** Clinical characteristics of included patients from databases (*n* = 1692).

Variable	Mean ± SD or subgroup	*n*
Age at diagnosis (years)	59.6 ± 11.5	1087
Disease-free survival (months)	22.2 ± 22.3	1192
Overall survival (months)	37.5 ± 29.6	1657
Clinical stage	IA	3
	IB	3
	IC	11
	IIA	7
	IIB	9
	IIC	41
	IIIA	15
	IIIB	46
	IIIC	780
	IV	168
Histologic grade	I	11
	II	204
	III	1323
	IV	2
	X	17
Race	White	919
	Black	64
	Asian	39
	American Indian	6
Fraction genome altered	0.56 ± 0.18	1653
Karnofsky performance score	75.98 ± 13.43	87
Longest dimension of lesion (cm)	1.38 ± 0.60	578
Lymphovascular invasion indicator	Yes	139
	No	85
Primary tumor site	Bilateral	412
	Left	85
	Right	72
Neoplasm status	Tumor free	267
	With tumor	684
Mutation count	63.70 ± 79.92	1041

**Table 2:** Summary of value of predictive factors for disease-free survival.

Variable	Cutoff	*p* value
Age at diagnosis (years)	75	0.021
Clinical stage	Overall	<0.001
	II	0.003
	IIC	<0.001
	IIIA	<0.001
	IIIB	<0.001
	IIIC	<0.001
	IV	<0.001
Histologic grade	Overall	0.01
	II	0.273
	III	0.014
Race	Overall	0.443
Fraction genome altered	Overall	>0.05
Karnofsky performance score	Overall	0.327
Longest dimension of lesion (cm)	3	0.007
Lymphovascular invasion indicator	Overall	0.346
Primary tumor site	Overall	0.438
Neoplasm status	Overall	<0.001
Mutation count	30	0.004
	50	<0.001

**Table 3:** Summary of value of predictive factors for overall survival from database.

Variable	Cutoff	*p* value
Age at diagnosis (years)	Overall	<0.001
	50	0.003
	60	<0.001
	70	<0.001
	75	<0.001
Clinical stage	Overall	0.121
	II	0.201
	IIC	0.017
	IIIA	<0.001
	IIIB	<0.001
	IIIC	<0.001
	IV	<0.001
Histologic grade	Overall	0.009
	II	0.222
	III	0.037
Race	Overall	0.004
Fraction genome altered	0.4	0.032
	0.5	0.03
	0.6	0.001
Karnofsky performance score	Overall	0.37
Longest dimension of lesion (cm)	3	0.091
Lymphovascular invasion indicator	Overall	0.064
Primary tumor site	Overall	0.825
Neoplasm status	Overall	<0.001
Mutation count	10	<0.001
	20	<0.001
	30	<0.001
	50	<0.001

**Table 4:** Summary of value of predictive factors for overall survival from admitted patients.

Variable	Cutoff	*p* value	No. of patients
Age at diagnosis (years)	Overall	0.224	38
	40	0.203	
	50	0.333	
	60	0.065	
Histologic grade	Overall	0.155	38
	II	0.091	
Race	Han	0.846	38
Longest dimension of lesion (cm)	Overall	0.001	36
	4	0.933	
	6	0.912	
	8	0.842	
Primary tumor site	Left, right, and both	0.158	38
Body weight (kg)	Overall	<0.001	38
	50	0.184	
	60	0.566	
Neoplasm status	Lesion removed	0.116	38
Treatment	Surgery, chemotherapy, or both	0.15	38
Live with family	Yes or no	0.551	38

## Data Availability

The datasets generated during and/or analyzed during the current study are available from the corresponding author on reasonable request.
